# Intra- and Interpersonal Dimensions of Orthorexia: Preliminary Development and Validation of an Intra- and Interpersonal Effects Scale for Orthorexia

**DOI:** 10.3390/nu16071010

**Published:** 2024-03-29

**Authors:** Wojciech Styk, Mateusz Gortat, Emilia Samardakiewicz-Kirol, Szymon Zmorzynski, Marzena Samardakiewicz

**Affiliations:** 1 Academic Laboratory of Psychological Tests, Medical University of Lublin, 20-059 Lublin, Poland; 2 Institute of Education and Practical Improvement, Association of Young Scientists in Poland, 20-560 Lublin, Poland; 3 Department of Didactics and Medical Simulation, Medical University of Lublin, 20-059 Lublin, Poland; 4 Laboratory of Genetics, Academy of Zamość, 22-400 Zamość, Poland; 5 Department of Psychology, Medical University of Lublin, 20-059 Lublin, Poland

**Keywords:** ortorexia nervosa, eating disorders, TOS, IIESO

## Abstract

Orthorexia nervosa (ON) is a disorder characterized by dietary restrictions and an obsessive focus on “healthy” eating. The present study analyzes two aspects of ON. One related to the inner experiences of the individual (intrapersonal). The other concerns the impact of ON on interpersonal relationships (interpersonal). The developed scale was named the Intra- and Interpersonal Effects Scale of Orthorexia (IIESO). The analysis showed an average correlation between the INTER and INTRA factors (r = 0.46). Both the INTER and INTRA scales correlated strongly with both subscales of the TOS but weakly with the ORTO-R score. Females obtained higher scores on the INTER scale (*p* < 0.01), while no differences were shown for the INTRA subscale or the overall scale score (*p* < 0.01). Subjects using supplements had higher mean scores on the INTER and INTRA subscales and for the total score. Among the analyzed results, the greatest strength effect was shown for the total score on the IIESO scale (INTER+INTRA) and the TOS scale. The questionnaires used to date have not distinguished between behaviors from interpersonal and intrapersonal perspectives. Research on these dimensions could expand our knowledge of the disorder and refine diagnostic criteria.

## 1. Introduction

Although more than a quarter of a century has passed since the publication of the first paper on the topic of orthorexia nervosa (ON), diagnostic criteria for this disorder have still not been developed. Reliable tools to measure the variables associated with the disorder have not yet been created to this date [1]. ON is described as a disorder in which there is an obsessive focus on “healthy” eating and dietary restrictions. This focus is very broad and occurs at the intersection of social influences and daily life psychopathology [2]. In a review paper, Saraswati et al. described orthorexia as an obsession with avoiding foods perceived as unhealthy, often leading to emotional disturbances and restrictive eating patterns [3]. In contrast, Kalra et al. emphasized the clinical features of this disease, noting the bidirectional links between endocrinopathy and orthorexia [4]. Such different approaches, and indeed, the lack of one consistent one, are reflected in the ON measurement tools used. A systematic review by Valente et al. indicates that the most commonly used are the Bratman Orthorexia Test (BOT), the Questionnaire for the Diagnosis of Orthorexia (ORTO-15), the Eating Habits Questionnaire (EHQ), the Düsseldorf Orthorexia Scale (DOS), the Barcelona Orthorexia Scale (BOS), and the Teruel Orthorexia Scale (TOS) [5]. The BOT scale is based on the concept of an obsession with healthy eating, which seems to acquire features of the ED [6]. The ORTO-15 and ORTO-R scales, on the other hand, are based on the conceptualization of ON as abusive eating behavior, characterized by a combination of eating, behavioral, and obsessive-phobic personality traits [7]. The EHQ scale speaks of an overwhelming preoccupation with healthy eating, and the DOT and BOS scales are based on the concept of fixation on healthy eating [5]. And finally, the TOS scale describes extreme or excessive preoccupation with eating foods considered healthy [8]. The tools described are based on different conceptualizations of ON and diagnostic criteria, but, importantly, all conceptualizations were derived from Bratman’s initial definition of ON in 1997 [5].

Another approach described ON as an obsessive-compulsive disorder that requires special care in choosing “clean” and healthy foods, leading to a restrictive diet and social isolation [9,10]. In clinical practice, several attempts have been made for several years to apply diagnostic criteria for orthorexia. These criteria differ from each other. Nevertheless, an attempt can be made to standardize them. The basic diagnostic criteria for orthorexia include (a) obsessive or pathological preoccupation with healthy eating; (b) emotional consequences of noncompliance with self-imposed dietary rules (e.g., suffering, anxiety); and (c) psychosocial impairments in selected life areas, as well as malnutrition and weight loss [11].

Several risk factors for orthorexia have been described. One of them was young. The authors showed that younger people have a greater risk of ON. This is probably due to a greater preoccupation with physical attractiveness during this period of life. Younger people also often introduce alternative forms of eating, putting them at significant risk of developing abnormal eating behaviors [12,13]. It was also found that people with an abnormal BMI—both above and below the normal range—are more likely to develop ON than patients with other disorders, such as inflammatory bowel disease [14].

Syurina et al. believe that ON is a dietary pattern that occurs mainly in Western countries. Higher wealth levels make it easier to access healthy and organic foods [11]. The diet industry and the fitness industry have had a tremendous impact on the development of an obsession with healthy food, the pursuit of the ideal slim figure, and the desire to control one’s body [1,15]. Social media also has a significant impact on orthorexic behavior creation. Often, a person at risk of orthorexia receives approval for his or her actions on the Internet, which reaffirms them [16,17,18]. Initial research on ON also suggested the presence of specific risk groups. People in professions that are associated with care for health and appearance, i.e., artists, athletes, and representatives of the broader cultural world, are classified in a high-risk group for orthorexia. This group also includes people who care about the health of others, such as medics, nutritionists, and fitness trainers. Additionally, certain dietary choices or lifestyles, such as vegetarianism and veganism, may contribute to an increased risk of orthorexia [13,17,18,19]. The study showed that the incidence of ON varies from country to country, ranging from 6.9% in the Italian population to 88.7% in a group of Brazilian diet students, indicating that some groups appear to be more susceptible to the risk of ON development than others [20].

The strong psychological component of the disorder is associated with difficulty regulating emotions and negative affect, as well as eccentric beliefs [21,22]. Social isolation is also a consequence of this disorder. The studies described orthorexia as a process that leads to a restrictive diet and social isolation as compensation. These findings indicate that orthorexia may contribute to social isolation and poorer social functioning [10,23,24]. In addition, individuals with orthorexia may experience reduced pleasure during social gatherings or communal meals due to the stress and anxiety associated with dietary choices, again leading to social withdrawal and feelings of alienation. As the disorder progresses, individuals may develop distorted beliefs about the relationship between diet and health, leading to rigid thinking and impaired cognitive flexibility [25]. Because people with ON prioritize their dietary and health-focused restrictions, they may additionally experience increased anxiety and stress in social situations that involve food, leading them to avoid social gatherings or events centered around meals [22]. Ultimately, the psychological toll of ON can significantly affect a person’s emotional well-being, interpersonal relationships, social functioning, and overall quality of life [26,27]. ON impacts various aspects of an individual’s daily life and can include a relentless preoccupation with food purity and rigid dietary rules, which can dominate one’s thoughts and behaviors, leading to a reduced ability to engage in social activities and avoid the presence of others during food consumption [28]. The resulting social isolation and withdrawal can, in turn, negatively affect interpersonal relationships and make it difficult to develop meaningful relationships with others. Obsession about “clean eating” can also extend to family members or friends, creating tension when others do not follow the same dietary rules. The profound impact of ON on social functioning can contribute to feelings of loneliness, depression, and reduced well-being [27]. Orthorexia negatively affects physical, mental, and social health. Additional research on valid and reliable screening instruments, body image, and psychological functioning would help to better understand the disorder and expand treatment options [29].

As we have indicated, although orthorexia is not officially recognized as a separate disease in the classification of disorders, it is still an area of research and discussion in the field of mental health and nutrition. The lack of diagnostic criteria makes it necessary to study the phenomenon in depth, for which appropriate tools are needed. In this study, we hypothesized two aspects of ON. One related to the inner experiences of the individual (intrapersonal) concerning the feelings, desires, and beliefs of the individual. The other concerns the impact of ON on interpersonal relationships (interpersonal). Currently, there is no research tool that assesses ON in these two dimensions. Therefore, the purpose of this study was to develop a scale to capture these two dimensions of orthorexic behavior and study the proposed construct.

## 2. Methods

The aim of this study is to develop a questionnaire covering two dimensions not yet studied in relation to orthorexic behavior, i.e., the intra- and interpersonal dimensions. Another goal is to analyze the proposed construct. For this purpose, the validated psychological measurement tools described below were used.

### 2.1. Instruments

#### 2.1.1. The Teruel Orthorexia Scale by Barrada and Roncero (TOS)

The questionnaire was validated using the guidelines included in the document describing the translation and adaptation of WHO instruments [30,31]. The Polish adaptation of the TOS scale consisted of 17 statements. Individual questions in the TOS can be answered on a 5-point scale, where 1 means the statement “does not concern you at all”, 5 means the statement “completely affects you”, and 3 means a neutral attitude. The scale was divided into two subscales: TOS He, healthy orthorexia without a pathological carrier; and TOS Ne, pathological/negative orthorexia [8]. The reliability of both scales obtained in the original version and translations had a value of at least 0.8. In our sample, the reliability was 0.82 for TOS Ne and 0.87 for TOS He.

#### 2.1.2. The ORTO-R Questionnaire by Radosław Rogoza and Lorenzo M. Donini

The ORTO-R is based on a frequently used ORTO-15, overcoming its main limitation of an unstable factorial structure across different populations [7]. It consists of 6 questions. The answers can be given on a 5-point scale. The revised version has better psychometric properties. Moreover, a univariate structure with good reliability has been confirmed in large sample studies [29]. In our sample, the reliability of the scale as measured by Cronbach’s alpha coefficient was 0.91.

The KZZJ questionnaire (Polish: Kwestionariusz Zachowań Związanych z Jedzeniem), developed by Ogińska-Bulik and Putyński was used to determine eating behavior [30]. The KZZJ was developed based on the Eating Disorder Inventory and the Eating Attitude Test. The KZZJ consists of 30 items that make up the 3 scales of the questionnaire: Habitual Overeating (KZZJ_HO), Emotional Overeating (KZZJ_EO), and Diet Restrictions (KZZJ_DR) [32]. The test allows us to examine the tendency to overeat or refrain from eating. It is recommended to assess the risk of overweight or obesity in normal-weight individuals and to observe progress during obesity treatment. According to the authors, the scale can be used across age groups in obese and nonobese individuals, as well as in individuals with eating disorders. In our sample, the reliability of the scale as measured by Cronbach’s alpha coefficient was 0.81 for KZZJ_HO, 0.84 for KZZJ_EO, and 0.79 for KZZJ_DR.

#### 2.1.3. The Body Esteem Scale (BES) by S. Franzoi and S. Shields

In this study, the Polish adaptation by M. Lipowska and M. Lipowski was used [31]. It consists of 35 test items in three subscales. Subscales for women are as follows: AS, sexual attractiveness; KW, weight control; and KF, physical conditioning. Subscales for men include AF—physical attractiveness; SC—body strength; and KF—physical conditioning [33]. To analyze the results, we used the overall scale score without analyzing the subscale scores. The reliability of the original version, measured by Cronbach’s alpha, ranged from 0.80 to 0.89, and in our sample, it was 0.81.

#### 2.1.4. Body Mass Index (BMI)

Body mass index (BMI) is a value derived from the mass (weight) and height of a person. BMI was defined as the body mass divided by the square of the body height and is expressed in units of kg/m^2^ [34]. The major adult BMI classifications were underweight (under 18.5 kg/m^2^), normal weight (18.5 to 24.9), overweight (25 to 29.9), and obese (30 or more) [35]. The subjects’ BMI was calculated based on their declared height and weight.

### 2.2. Participants and Procedures for Tool Development

**Figure 1:**
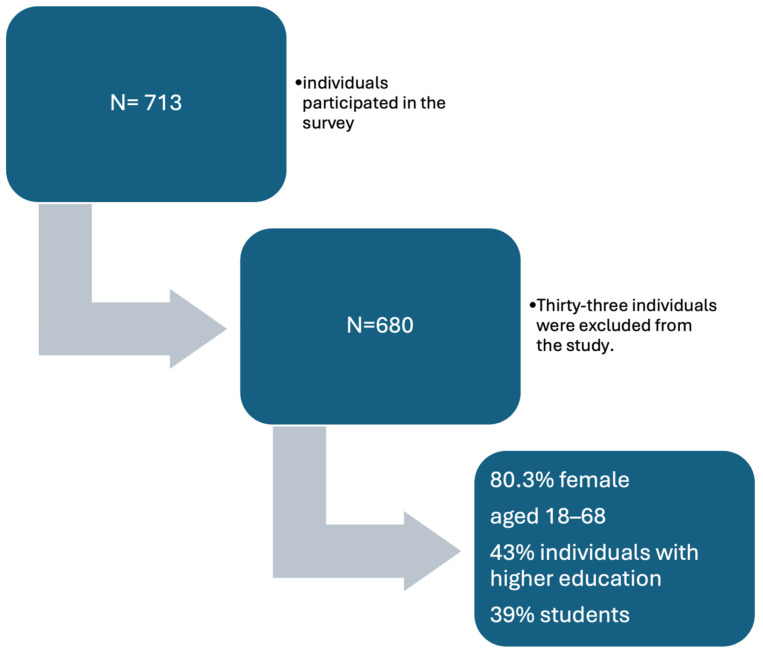
The flowchart describing the selection of the study sample.

The study was approved by the Bioethics Committee at the Medical University of Lublin (consent no. KE-0254/223/2019). The data were collected using self-reported measures. The survey was administered at universities and workplaces. The questionnaire was anonymous, and the respondents completed it on their own. A total of 713 individuals participated in the survey. After verification, 680 (80.3% female) questionnaires were included in further analysis. Thirty-three individuals were excluded from the study due to their age being too low (<18) or because of incomplete questionnaires. The survey also collected sociodemographic and anthropometric data, such as weight and height, use of supplements, and applied diets. In the present study, the youngest person was 18 years old, and the oldest was 68 years old. The largest part of the study group consisted of individuals with higher education (43%) and, in turn, students (39%). Forty-seven percent of the study participants were working in the medical profession. The next largest occupational group in terms of size was individuals associated with humanistic professions (20%). Nearly 28% of the respondents declared that they were in college. The vast majority of respondents lived in a city with a population of more than 100,000 (47%). Forty-six percent of the respondents were in a rather good financial situation, and 41% were in an average financial situation. The flowchart describing the selection of the study sample is presented in Figure 1.

**Figure 2:**
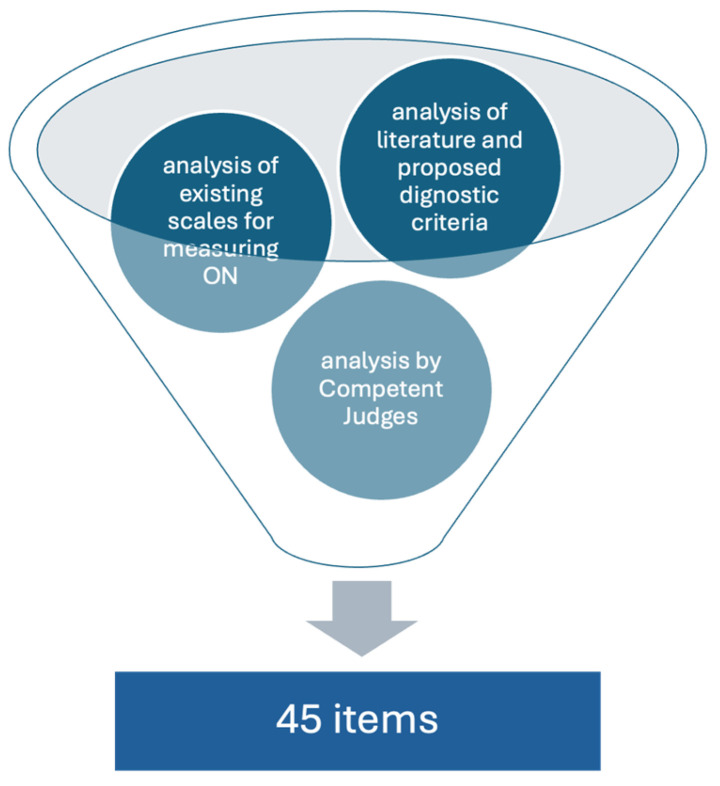
The procedure for developing the items.

The procedure for developing the items was based on a methodology using expert judges and panels of experts. Our goal was to create a tool that fits into the contemporary understanding of ON but would also be understood by a wide range of patients of different ages. First, an analysis of the literature describing proposals for ON diagnostic criteria was conducted. The scales used to date and the items used were also analyzed. The formulated statements were presented for selection to a group of several psychologists, who made a statement that most accurately described ON behavior. As a result of our work, 45 statements were selected, forming the items of the original scale. The original scale, developed in this way, was given to the respondents. The flowchart describing the procedure for developing the items is presented in Figure 2.

### 2.3. Statistical Analysis

To test our hypotheses, we conducted statistical analyses using JASP 0.17 software. An exploratory factor analysis (EFA) and a confirmatory factor analysis (CFA) were conducted to emerge the test structure and confirm the results, respectively. Basic descriptive statistics were calculated, and the Shapiro–Wilk normality test was applied. Correlation analysis was performed using Pearson’s r coefficient. The values of variables compared in two groups were analyzed by the Student’s *t* test and in three groups by the analysis of variance (ANOVA), followed by Tukey’s post-hoc tests. The results were considered significant at *p* < 0.05.

## 3. Results

In the first step, an exploratory analysis of the collected data was carried out. The analysis identified two main factors associated with ON. In the next step, the items most strongly loading the extracted factors were extracted. A reliability analysis was also performed. As a result, 10 items for each factor were selected for the final scale version. Content analysis of the factors revealed that the first main factor was an interpersonal ON factor (INTER) and the second main factor was an intrapersonal ON factor (INTRA). This solution accounts for 40.7% of the explained variation. The findings from these analyses for the final scale are shown in Table 1.

In the next step, the results were subjected to confirmatory factor analysis (CFA). First, the KMO sample adequacy test and Bartlett’s sphericity test were applied. The KMO value was 0.75, indicating an adequate sample. The result of Bartlett’s sphericity test was statistically significant (χ^2^ = 1283.71, *p* < 0.001), which formed the basis for the factor analysis. The CFA showed a good fit of the model (GFI = 0.95; RMSEA = 0.75) consisting of two factors—INTER and INTRA. The loads specified in the CFA are shown in Table 2.

The developed scale was named the Intra- and Interpersonal Effects Scale of Orthorexia (IIESO). The scale allows the determination of a score on the INTRA subscale, referring to internal experiences related to orthorexic behavior, and the INTER subscale, referring to orthorexic behavior related to contact with other people and the community environment. The sum of these scores constitutes the total score of the IIESO scale. The final version of the IIESO can be found in Appendix A.

**Figure 3:**
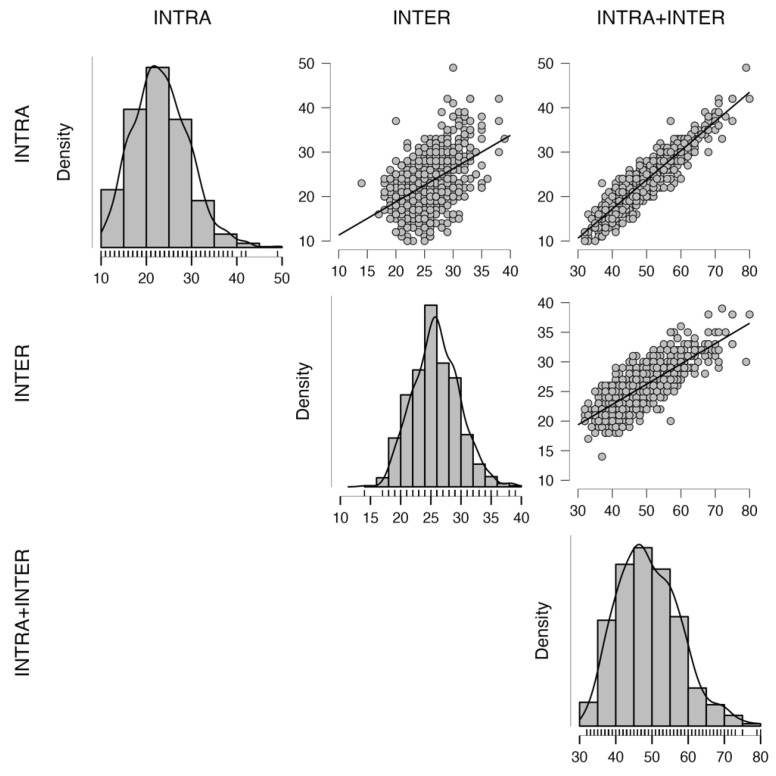
Density and correlation of variables. Black line indicates correlations of scales, gray dots represent analyzed cases.

In the next step, analyses were performed to determine the properties of the developed scale. For this purpose, a correlation analysis with the other variables was conducted. The analysis showed an average correlation between the INTER and INTRA factors (r = 0.46). Graphical analyses of these correlations and the distribution of the obtained results for the studied scales are presented in Figure 3.

Both the INTER and INTRA scales correlated strongly with both subscales of the TOS but weakly with the ORTO-R score. Weak correlations were also observed with the KZZJ scale. The results of the analyses are shown in Table 3.

A comparative analysis of the results obtained with two age groups (I) up to 30 years and (II) over 30 years was also carried out. An analysis of the differences between the correlations and a comparative analysis of the mean values of the variables studied were carried out. Statistically significant differences were observed in the studied groups, which may indicate different factors co-occurring with ON symptoms depending on the age group. The results are shown in Table 4 and Table 5.

**Figure 4:**
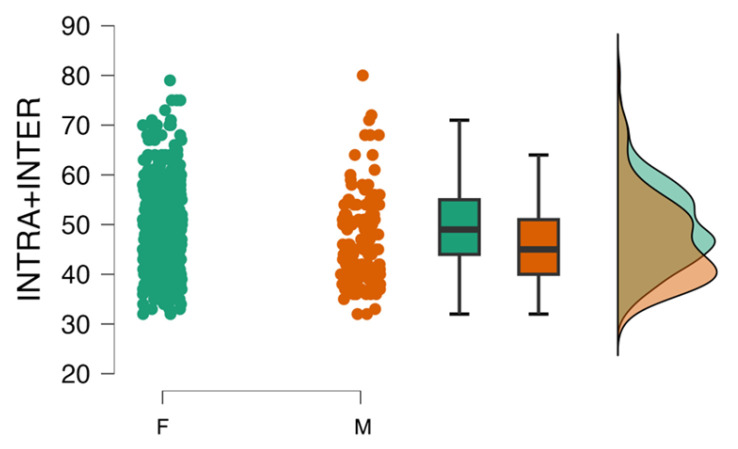

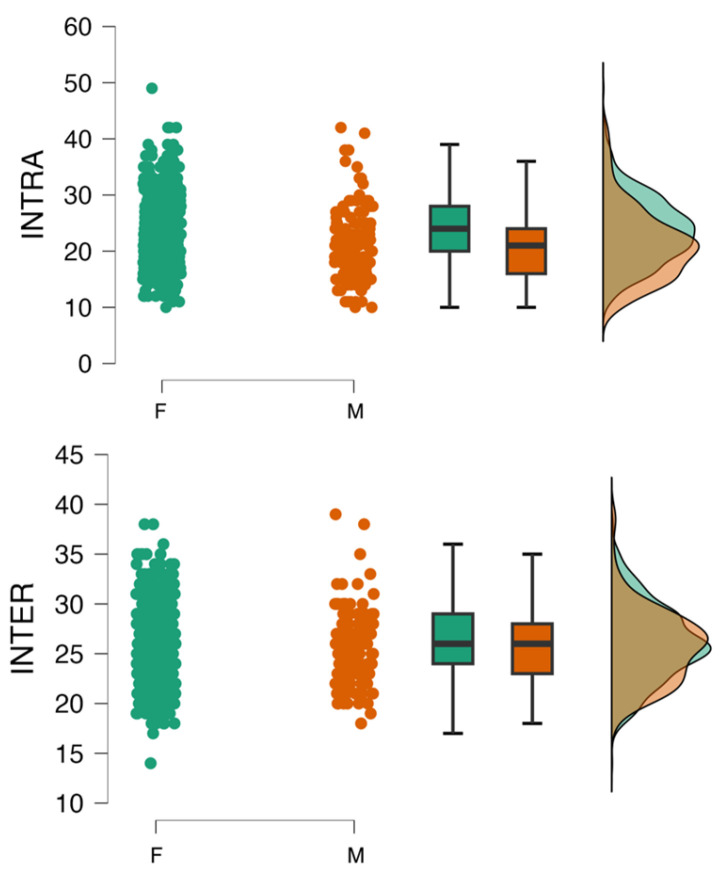
Distribution of INTRA and INTER variables according to sex (green for Female, orange for Male).

A comparative analysis of the obtained results was also carried out in terms of the subjects’ sex. Females obtained higher scores on the INTER scale (*p* < 0.01), while no differences were shown for the INTRA subscale or the overall scale score (*p* < 0.01). Analogous results were obtained for the ORTO-R and TOS_He scales; however, these scales had a lower strength of effect. An inverse relationship was obtained for the BES scale and for BMI. The results are presented in Table 6. The distribution of variables by sex for the INTER and INTRA personal scales studied is presented in Figure 4.

The subjects were also compared in terms of their declared use of dietary supplements. Subjects using supplements had higher mean scores on the INTER and INTRA subscales and for the total score. A similar relationship was observed for both subscales of the TOS scale and for the ORTO_R scale, but the strength of the effect was greatest for the INTRA scale. The results of the analyses are presented in Table 7.

The mean scores obtained in groups with different durations of diet experience were analyzed. The greatest differences were shown between study subjects who had never been on a diet and those who were currently on a diet. Among the analyzed results, the greatest strength effect was shown for the total score on the IIESO scale (INTER+INTRA) and the TOS scale. The results of the analyses are shown in Table 8.

## 4. Discussion

The purpose of our work was to develop a questionnaire covering two dimensions not yet studied in relation to orthorexic behavior, namely, the intra- and interpersonal dimensions. The degree of orthorexia in society still raises many questions. Numerous studies indicate the prevalence of this problem, but there is no consensus data on the severity of orthorexia. The prevalence of ON ranges from 6% to as high as 90% in the general population. Such significant variation may be due to cultural reasons or a defect in the measurement tool used [36,37]. Attempting to create accurate psychometric tools that describe various aspects of ON is important for characterizing the disorder and for more accurate diagnosis and treatment. The developed tool provides insight into the previously untapped inter- and intrapersonal spheres of ON-related behavior impact. The presented analyses testify that the tool is pertinent and reliable and can be the basis for further research into the ON phenomenon.

Orthorexia nervosa is not an entity included in international classifications of disorders (ICD-10/ICD-11 or DSM-5). In clinical practice, it is not clear whether orthorexia should be classified as an eating disorder or an obsessive-compulsive disorder. Because the symptoms involve the consumption of food, orthorexia can be categorized as a nonspecific eating behavior [38,39]. Therefore, during our research, we did not attempt to define norms or establish a cutoff point for maladaptive behavior, which would already indicate a disorder. In its current form, the scale is a tool that measures the level of ON’s impact on these two spheres of human functioning. The creator of the orthorexia concept modified his original concept and, in a 2017 publication, suggested the need to separate so-called healthy orthorexia, which applies to people who are interested in healthy eating but who do not have pathological effects on this behavior [6]. The TOS questionnaire, which makes it possible to distinguish between pathological orthorexia (mental/nervous) and healthy orthorexia, has become the answer to this demand [40]. The developed IIESO scale showed a strong correlation with both dimensions described by the TOS scale. Only the interpersonal dimension correlated moderately with healthy orthorexia. This confirms our intention, in which we did not focus on defining a catalog of healthy/unhealthy behaviors versus their consequences. The weaker strength of the correlation with the interpersonal dimension may be due to the small number of studies in this area and the classification of this behavior as abnormal. This indicates the need for further research on these dimensions and possibly the development of norms for the severity of individual traits in the future. An important result obtained in this study was the demonstration that different variables co-occur with ON symptoms depending on the age group studied (under and over 30). This trend should also be further studied.

Eating disorders are significantly more common in women than in men. However, there are no significant sex differences in the incidence of orthorexia [33,41]. Our study showed higher mean values for the INTRA scale and the IIESO scale total score among female respondents than among male respondents. The results of the INTER scale showed no differences between the sexes. Interestingly, our study group also showed differences by sex in the mean scores on the TOS_He and ORTO-R scales. However, the greatest strength of the effect of these differences was on the INTER scale, indicating that this scale was more effective at capturing sex-related differences. This is an important finding, indicating the need for further research related to this factor, which may suggest qualifying ON for eating disorders.

In our study, we also evaluated the associations between diet and supplement intake and the study variables. The analyses conducted showed that the mean values on the scales describing the phenomenon of orthorexia were greater for individuals who used supplements. The strength of this relationship was greatest for the TOS_Ne and IIESO scales. The results of our work are consistent with those of other studies [42,43]. Many authors note that vegetarian and vegan diets are particularly predisposed to orthorexia. Among Italian students in whom ON was present in 34.9% of cases, vegan and vegetarian diets were risk factors for orthorexia. Orthorexia symptoms were significantly more common in these groups than in those who preferred a traditional diet (56.2% vs. 32.2%) [44]. Domingues and Carmo, in their study, showed that the main predictor of orthorexia is the pursuit of a slim figure and following a healthy diet [45]. Our research also revealed a relationship between orthorexia symptoms and the use of dietary supplements. The greatest strength of this relationship was observed when the INTRA scale was tested. These results confirm reports from other studies that have shown a significant relationship between the use of dietary supplements and orthorexia development [46,47]. Oberle et al. studied individuals divided into three groups. The first group included individuals with orthorexia symptoms; the second group included individuals with a healthy diet; and the third group included individuals who were eating standard nutrition (these individuals comprised the control group). Their study revealed that people with orthorexia were significantly more likely to use dietary supplements than people in the other two study groups [48].

A distorted body image plays an important role in the onset of eating disorders [49]. Brytek-Matera suggested that people with orthorexia do not display a negative attitude toward their body image, which distinguishes them from those suffering from anorexia or bulimia [50]. People prone to orthorexic behavior are driven by concerns about their health rather than appearance issues [51]. Our results showed no significant correlation with body image. It should be noted that there have been few studies devoted to the relationship between orthorexia and the perception of one’s body, and this topic still needs to be thoroughly researched. The present study also analyzed eating behaviors as measured by the KZZJ questionnaire versus scores on various scales of orthorexia. The average correlations were found mainly with the Dietary Restrictions subscale and, interestingly, partly with the Emotional Overeating subscale. Dietary restraint, used as a measure against overweight, can paradoxically become a cause of overweight, as abstaining from food can lead to overeating [51]. Therefore, the relationship between the studied variables and emotional overeating is important. This type of behavior may not only be part of the fight against excess weight but also be related to tension relief and may be considered a method of stress reduction [51]. In contrast, the occurrence of food restriction is considered to be strongly associated with orthorexia [6,9,48,51].

The beginning of the new century resulted in the publication of a number of studies regarding unfavorable dietary behavior, which is considered one of the most important factors shaping health. Increased consumption of highly processed foods characterized by an inadequate balance of nutrients, as well as a lack of regular physical activity, has contributed to the deterioration of public health. One of the phenomena is presented in this work. Our hypothesis that it is possible to approach symptom measurement from an inter- and intrapersonal perspective was confirmed. This study does not exhaust the knowledge regarding the intrapersonal and interpersonal effects of orthorexia and requires further in-depth analysis.

## 5. Limitations

The main limitation in research on ON, and therefore in our study, is the lack of consistent diagnostic criteria. In recent years, work on orthorexia has been conducted at various research centers around the world. Currently, the proposed diagnostic criteria differ slightly from each other but are still not unambiguous. Further research is needed to help unify these findings and identify the right tools to capture the broad dimensions of this phenomenon. Since there are no official diagnostic criteria for ON, it was impossible to determine which participants had ON and treat them as a clinical group. In addition, we did not control our participants for any comorbidities other than ON. However, ON is not an official diagnosis, and there are no patient databases or other official records from which a representative sample can be obtained. Therefore, these results may not be representative of all individuals with ON. Another limitation is the performance of the analyses. EFA and CFA were carried out on the same sample. In subsequent studies, it is advisable to confirm the model on other probes, which will allow, among other things, a greater generalization of the results. As a limitation, it should also be pointed out that the survey sample was largely comprised of women, and the representation of men was significantly lower. Therefore, analyses conducted on the whole group may be more relevant for women than for men. Nevertheless, the presented questionnaire meets the criteria for psychometric measurement tools and examines two aspects related to orthorexic behavior. The questionnaires used to date have not distinguished between behaviors from interpersonal and intrapersonal perspectives. Research on these dimensions could expand our knowledge of the disorder and refine diagnostic criteria.

## 6. Conclusions

The Intra- and Interpersonal Effects Scale of Orthorexia Questionnaire is a reliable tool for measuring the severity of orthorexic behavioral effects. Research on ON that includes these two dimensions may provide new knowledge regarding this disorder. The analysis showed an average correlation between the INTER and INTRA factors. Both the INTER and INTRA scales correlated strongly with both subscales of the TOS but weakly with the ORTO-R. Females obtained higher scores on the INTER scale, while no differences were shown for the INTRA subscale or the overall scale score. Different scale scores have been shown depending on dietary stance and supplement intake.

## Figures and Tables

**Table 1:** Exploratory factor analysis.

Item No.	Content	INTRA	INTER	α Cr/% Explained Variance	
16	(EN) I am healthy because I avoid foods containing preservatives and artificial colors. (PL) Jestem zdrowy, bo unikam zywności zawierającej konserwanty i sztuczne barwniki.	0.736		0.832/21.875%	0.827/40.707%
8	(EN) I often cannot sleep because I think about whether the meals I eat are safe and healthy. (PL) Często nie mogę zasnąć, bo myślę czy posiłki, które spożywam są bezpieczne i zdrowe.	0.724			
10	(EN) When I accidentally eat something unhealthy, I think about inducing vomiting. (PL) Gdy zdarzy mi się zjeść coś niezdrowego myślę, żeby spowodować wymioty.	0.658			
11	(EN) I am overwhelmed by guilt when I eat something unhealthy. (PL) Prześladuje mnie poczucie winy, gdy zjem coś niezdrowego.	0.638			
2	(EN) When I eat an unhealthy meal, I deny myself the rest of the meals that day, even when they are healthy and I am hungry. (PL) Gdy zjem niezdrowy posiłek odmawiam sobie pozostałych posiłków tego dnia nawet gdy są one zdrowe a ja jestem głodna/y.	0.63			
9	(EN) When I eat something unhealthy, I follow a cleansing diet. (PL) Gdy zjem coś niezdrowego to stosuje diety oczyszczające.	0.624			
20 *	(EN) I do not pay much attention to the meals I eat. (PL) Nie przykładam większej uwagi do spożywanych posiłków.	0.586			
6	(EN) My knowledge about healthy eating and harmful substances in food is high. (PL) Moja wiedza na temat zdrowego odżywiania i szkodliwych substancji w żywności jest duża.	0.532			
19	(EN) I pay a lot of attention to meal planning and preparation. (PL) Poświęcam dużo uwagi planowaniu i przygotowywaniu posiłków.	0.474			
17	(EN) Food is of poor quality, so dietary supplements must be taken. (PL) Żywność jest złej jakości, dlatego trzeba zażywać suplementy diety.	0.454			
3	(EN) I try to make friends and family realize that it is necessary to carefully choose products to consume in order to be healthy. (PL) Staram się uświadamiać znajomych i rodzinę o tym, że trzeba dokładnie wybierać produkty do spożycia, aby były zdrowe.		0.729	0.783/18.832%	
15	(EN) I believe that others do not pay adequate attention to the quality and safety of the products that we consume, which can be dangerous to our health. (PL) Uważam, że inni nie przykładają odpowiedniej uwagi do jakości i bezpieczeństwa produktów które spożywamy co może być niebezpieczne dla zdrowia.		0.677		
12	(EN) Others think I place too much importance on a healthy lifestyle and nutrition. (PL) Inni uważają, że przykładam zbyt dużo wagi zdrowemu stylowi życia i odżywianiu się.		0.673		
1	(EN) Often, during conversations with friends, I discuss the importance of a healthy diet. (PL) Często podczas rozmów ze znajomymi opowiadam jak ważna jest zdrowa dieta.		0.642		
7	(EN) My friends consider me a person who has a lot of knowledge about healthy food. (PL) Znajomi uznają mnie za osobę, która ma dużą wiedzę na temat zdrowej żywności.		0.641		
14	(EN) I have the impression that my friends avoid talking to me about healthy eating. (PL) Mam wrażanie, że znajomi unikają ze mną rozmów na temat zdrowego odżywiania się		0.537		
18	(EN) During meetings with friends, we exchange knowledge about the harmfulness of certain foods. (PL) Podczas spotkań ze znajomymi wymieniamy się wiedzą na temat szkodliwości niektórych pokarmów.		0.534		
13 *	(EN) I often eat meals prepared by others. (PL) Często spożywam posiłki przygotowane przez inne osoby.		0.600		
5 *	(EN) I often order ready-made meals instead of cooking myself. (PL) Często zamawiam gotowe posiłki zamiast gotować samemu.		0.562		
4 *	(EN) I like to dine at restaurants/lounges. (PL) Lubię spożywać posiłki w restauracjach/lokalach.		0.562		

* Reversed item; (PL)—content in Polish; (EN)–content in English.

**Table 2:** Confirmatory factor analysis.

Factor		Indicator	Loadings
INTRA	16	(EN) I am healthy because I avoid foods containing preservatives and artificial colors. (PL) Jestem zdrowy, bo unikam zywności zawierającej konserwanty i sztuczne barwniki.	0.70
	8	(EN) I often cannot sleep because I think about whether the meals I eat are safe and healthy. (PL) Często nie mogę zasnąć, bo myślę czy posiłki, które spożywam są bezpieczne i zdrowe.	0.74
	10	(EN) When I accidentally eat something unhealthy, I think it will cause vomiting. (PL) Gdy zdarzy mi się zjeść coś niezdrowego myślę, żeby spowodować wymioty.	0.75
	11	(EN) I am persecuted by guilt when I eat something unhealthy. (PL) Prześladuje mnie poczucie winy, gdy zjem coś niezdrowego.	0.59
	2	(EN) When I eat an unhealthy meal, I deny myself the rest of the meals on that day, even when they are healthy and I am hungry. (PL) Gdy zjem niezdrowy posiłek odmawiam sobie pozostałych posiłków tego dnia nawet gdy są one zdrowe a ja jestem głodna/y.	0.65
	9	(EN) When I eat something unhealthy, I follow a cleansing diet. (PL) Gdy zjem coś niezdrowego to stosuje diety oczyszczające.	0.59
	20 *	(EN) I do not pay much attention to the meals I eat. (PL) Nie przykładam większej uwagi do spożywanych posiłków.	0.49
	6	(EN) My knowledge about healthy eating and harmful substances in food is high. (PL) Moja wiedza na temat zdrowego odżywiania i szkodliwych substancji w żywności jest duża.	0.52
	19	(EN) I pay a lot of attention to meal planning and preparation. (PL) Poświęcam dużo uwagi planowaniu i przygotowywaniu posiłków.	0.44
	17	(EN) Food is of poor quality, so dietary supplements must be taken. (PL) Żywność jest złej jakości, dlatego trzeba zażywać suplementy diety.	0.48
INTER	3	(EN) I try to make friends and family realize that it is necessary to carefully choose products to consume in order to be healthy. (PL) Staram się uświadamiać znajomych i rodzinę o tym, że trzeba dokładnie wybierać produkty do spożycia, aby były zdrowe.	0.77
	15	(EN) I believe that others do not pay adequate attention to the quality and safety of the products that we consume, which can be dangerous to our health. (PL) Uważam, że inni nie przykładają odpowiedniej uwagi do jakości I bezpieczeństwa produktów które spożywamy co może być niebezpieczne dla zdrowia.	0.73
	12	(EN) Others think I place too much importance on a healthy lifestyle and nutrition. (PL) Inni uważają, że przykładam zbyt dużo wagi zdrowemu stylowi życia i odżywianiu się.	0.71
	1	(EN) Often, during conversations with friends, I discuss how important a healthy diet is. (PL) Często podczas rozmów ze znajomymi opowiadam jak ważna jest zdrowa dieta.	0.49
	7	(EN) My friends consider me a person who has a lot of knowledge about healthy food. (PL) Znajomi uznają mnie za osobę, która ma dużą wiedzę na temat zdrowej żywności.	0.54
	14	(EN) I have the impression that my friends avoid talking to me about healthy eating. (PL) Mam wrażanie, że znajomi unikają ze mną rozmów na temat zdrowego odżywiania się	0.73
	18	(EN) During meetings with friends, we exchange knowledge about the harmfulness of certain foods. (PL) Podczas spotkań ze znajomymi wymieniamy się wiedzą na temat szkodliwości niektórych pokarmów.	0.68
	13 *	(EN) I often eat meals prepared by others. (PL) Często spożywam posiłki przygotowane przez inne osoby.	0.66
	5 *	(EN) I often order ready-made meals instead of cooking for myself. (PL) Często zamawiam gotowe posiłki zamiast gotować samemu.	0.48
	4 *	(EN) I like to dine in restaurants/lounges. (PL) Lubię spożywać posiłki w restauracjach/lokalach.	0.44

* Reversed item (PL)—content in Polish; (EN)–content in English.

**Table 3:** Pearson’s correlation analysis.

		INTRA	INTER	INTRA+INTER	TOS_He	TOS_Ne	ORTO-R	BES	KZZJ_HO	KZZJ_EO	KZZJ_DR	BMI
INTRA	Pearson’s r	—										
	Effect size (Fisher’s z)	—										
INTER	Pearson’s r	0.46 ***	—									
	Effect size (Fisher’s z)	0.50	—									
INTRA+INTER	Pearson’s r	0.92 ***	0.77 ***	—								
	Effect size (Fisher’s z)	1.59	1.03	—								
TOS_He	Pearson’s r	0.68 ***	0.30 ***	0.62 ***	—							
	Effect size (Fisher’s z)	0.84	0.31	0.73	—							
TOS_Ne	Pearson’s r	0.62 ***	0.53 ***	0.68 ***	0.58 ***	—						
	Effect size (Fisher’s z)	0.72	0.60	0.83	0.66	—						
ORTO_R	Pearson’s r	0.37 ***	0.27 ***	0.38 ***	0.43 ***	0.46 ***	—					
	Effect size (Fisher’s z)	0.39	0.27	0.40	0.46	0.50	—					
BES	Pearson’s r	0.01	−0.11 **	−0.05	0.13 ***	−0.08 *	−0.21 ***	—				
	Effect size (Fisher’s z)	0.01	−0.11	−0.05	0.13	−0.08	−0.22	—				
KZZJ_HO	Pearson’s r	−0.03	0.08 *	0.01	−0.06	0.16 ***	0.35 ***	−0.25 ***	—			
	Effect size (Fisher’s z)	−0.03	0.08	0.01	−0.06	0.16	0.36	−0.26	—			
KZZJ_EO	Pearson’s r	0.08 *	0.25 ***	0.17 ***	0.03	0.32 ***	0.37 b***	−0.35 ***	0.55 ***	—		
	Effect size (Fisher’s z)	0.08	0.26	0.17	0.03	0.33	0.39	−0.37	0.62	—		
KZZJ_DR	Pearson’s r	0.33 **	0.36 ***	0.39 ***	0.25 ***	0.49 ***	0.45 ***	−0.33 ***	0.24 ***	0.49 ***	—	
	Effect size (Fisher’s z)	0.34	0.37	0.41	0.26	0.53	0.48	−0.34	0.24	0.54	—	
BMI	Pearson’s r	0.01	0.04	0.02	−0.09 *	−0.04	−0.02	−0.19 ***	0.07	0.16 ***	0.17 ***	—
	Effect size (Fisher’s z)	0.01	0.04	0.02	−0.09	−0.04	−0.02	−0.19	0.07	0.16	0.17	—
Age	Pearson’s r	0.12 **	−0.03	0.07	0.11 **	−0.04	−0.10 *	0.01	−0.19 ***	−0.11 **	−0.03	0.37 ***
	Effect size (Fisher’s z)	0.12	−0.03	0.07	0.11	−0.04	−0.10	0.01	−0.19	−0.11	−0.03	0.38

* p < 0.05; ** *p* < 0.01; *** *p* < 0.001.

**Table 4:** Pearson’s correlation analysis and *p*-value of Fisher’s Z-test for comparing differences between age groups.

		AGE < 30 (n = 469)	AGE ≥ 30 (n = 211)	Fisher’s Z *p*
INTRA	INTER	0.50 ***	0.44 ***	0.18
	INTRA+INTER	0.93 ***	0.90 ***	0.01
	BMI	−0.05	0.01	0.32
	TOS_He	0.70 ***	0.63 ***	0.07
	TOS_Ne	0.66 ***	0.53 ***	0.08
	KZZJ_HO	−0.01	−0.05	0.32
	KZZJ_EO	0.13 **	0.01	0.07
	KZZJ_DR	0.37	0.22 **	0.02
	BES	−0.03	0.08	0.27
	ORTO_R	0.39 ***	0.37 ***	0.39
	AGE	−0.03	0.14 *	0.09
INTER	INTRA+INTER	0.78 ***	0.78 ***	0.05
	BMI	0.01	0.20 **	0.01
	TOS_He	0.37 ***	0.21 **	0.02
	TOS_Ne	0.57 ***	0.45 ***	0.03
	KZZJ_HO	0.06	0.09	0.36
	KZZJ_EO	0.26 ***	0.21 **	0.26
	KZZJ_DR	0.38 ***	0.31 ***	0.17
	BES	−0.11 *	−0.09	0.40
	ORTO_R	0.31 ***	0.15 *	0.02
	AGE	−0.06	0.23 ***	0.02
INTRA	BMI	−0.03	0.09	0.24
+INTER	TOS_He	0.66 ***	0.54 ***	0.01
	TOS_Ne	0.71 ***	0.58 ***	<0.01
	KZZJ_HO	0.02	0.01	0,45
	KZZJ_EO	0.20 ***	0.11	0,13
	KZZJ_DR	0.43 ***	0.30 ***	0,04
	BES	−0.07	0.01	0.24
	ORTO_R	0.41 ***	0.32 ***	0.11
	AGE	−0.05	0.20 **	0.03

* p < 0.05; ** *p* < 0.01; *** *p* < 0.001.

**Table 5:** Comparison between age groups.

	M (SD)		*p*	Cohen’s d
	<30	≥30		
INTRA	22.83 (6.39)	24.25 (5.48)	<0.01	−0.23
INTER	26.23 (3.81)	25.37 (3.77)	<0.01	0.23
INTRA+INTER	49.06 (8.92)	49.62 (7.91)	0.43	
TOS_He	26.47 (7.70)	28.46 (7.77)	<0.01	−0.26
TOS_Ne	15.61 (6.01)	15.04 (5.02)	0.23	
KZZJ_HO	4.26 (2.71)	3.28 (2.75)	<0.01	0.36
KZZJ_EO	4.63 (2.41)	4.11 (2.55)	0.01	0.21
KZZJ_DR	3.40 (2.59)	3.20 (2.59)	0.34	
ORTO_R	13.89 (3.20)	13.36 (3.17)	0.04	0.17
BES	230.67 (53.75)	234.04 (48.46)	0.44	
BMI	21.98 (3.47)	24.62 (4.78)	<0.01	−0.67

**Table 6:** Comparison of sex groups.

	Sex	n	Mean	SD	*p*	Cohen’s d
INTER	F	546	23.82	5.97	<0.01	0.46
	M	134	21.01	6.41		
INTRA	F	546	26.04	3.85	0.27	
	M	134	25.63	3.66		
INTER+INTRA	F	546	49.86	8.43	<0.01	0.38
	M	134	46.65	8.91		
TOS_He	F	546	27.48	7.57	<0.001	0.26
	M	134	25.49	8.37		
TOS_Ne	F	546	15.48	5.75	0.67	
	M	134	15.25	5.61		
ORTO_R	F	546	13.90	3.09	<0.01	0.28
	M	134	13.02	3.55		
KZZJ_HO	F	546	3.98	2.80	0.69	
	M	134	3.87	2.57		
KZZJ_EO	F	546	4.46	2.43	0.79	
	M	134	4.52	2.61		
KZZJ_DR	F	546	3.44	2.60	0.05	0.19
	M	134	2.95	2.28		
BES	F	546	229.54	50.43	0.03	0.21
	M	134	240.59	58.01		
BMI	F	546	22.58	4.12	<0.01	0.27
	M	134	23.70	3.95		
Age	F	546	28.27	10.06	0.16	
	M	134	26.94	8.37		

**Table 7:** Comparison of supplement use groups.

	Supp *	n	Mean	SD	*p*	Cohen’s d
INTRA	No	259	21.29	6.06	<0.01	0.54
	Yes	421	24.48	5.90		
INTER	No	259	25.44	3.49	<0.01	0.22
	Yes	421	26.29	3.97		
INTRA+INTER	No	259	46.73	8.02	<0.01	0.48
	Yes	421	50.77	8.62		
TOS_He	No	259	24.78	7.67	<0.001	0.49
	Yes	421	28.51	7.49		
TOS_Ne	No	259	14.17	4.66	<0.001	0.36
	Yes	421	16.21	6.16		
ORTO_R	No	259	13.14	3.18	<0.001	0.30
	Yes	421	14.09	3.16		
KZZJ_HO	No	259	4.14	2.90	0.18	
	Yes	421	3.85	2.66		
KZZJ_EO	No	259	4.35	2.46	0.32	
	Yes	421	4.54	2.47		
KZZJ_DR	No	259	2.92	2.30	<0.001	0.27
	Yes	421	3.60	2.65		
BES	No	259	229.60	52.32	0.41	
	Yes	421	233.02	52.07		
BMI	No	259	23.03	4.36	0.09	
	Yes	421	22.66	3.94		
Age	No	259	27.85	9.58	0.76	
	Yes	421	28.10	9.87		

* use of nutritional supplements.

**Table 8:** Comparison of different diet use groups.

		N	Mean	SD	Comparison	*p*	Cohen’s d
INTRA	1. I am on a diet	71	26.13	6.59	1-2	0.01	0.37
	2. I have been on a diet	371	23.94	5.93	1-3	<0.001	0.80
	3. I have never been on a diet	238	21.37	5.83	2-3	<0.001	0.43
INTER	1. I am on a diet	71	27.07	3.91	1-2	0.42	
	2. I have been on a diet	371	26.46	3.75	1-3	<0.001	0.59
	3. I have never been on a diet	238	24.85	3.64	2-3	<0.001	0.43
INTER+INTRA	1. I am on a diet	71	53.20	9.47	1-2	0.03	0.34
	2. I have been on a diet	371	50.40	8.37	1-3	<0.001	0.84
	3. I have never been on a diet	238	46.22	7.79	2-3	<0.001	0.50
TOS_He	1. I am on a diet	71	31.07	7.69	1-2	<0.001	0.46
	2. I have been on a diet	371	27.59	7.36	1-3	<0.001	0.79
	3. I have never been on a diet	238	25.12	7.88	2-3	<0.001	0.33
TOS_Ne	1. I am on a diet	71	18.97	7.02	1-2	<0.001	0.54
	2. I have been on a diet	371	15.99	5.68	1-3	<0.001	0.98
	3. I have never been on a diet	238	13.51	4.57	2-3	<0.001	0.45
ORTO-R	1. I am on a diet	71	14.94	3.20	1-2	0.09	
	2. I have been on a diet	371	14.09	3.17	1-3	<0.001	0.69
	3. I have never been on a diet	238	12.80	3.01	2-3	<0.001	0.41

## Data Availability

Data are contained within the article.

## Abbreviations

BES	The Body Esteem Scale
BMI	Body mass index
IIESO	The Intra- and Interpersonal Effects Scale of Orthorexia
INTER	Interpersonal subscale score
INTRA	Intrapersonal subscale score
INTER+INTRA	IIESO scale total score
KZZJ_HO	Habitual Overeating subscale
KZZJ_EO	Emotional Overeating subscale
KZZJ_DR	Diet Restrictions subscale
ON	Orthorexia nervosa
TOS	Toruel Ortorexia Scale
TOS_Ne	subscale of the TOS scale, examining the severity of orthorexia, referred to as nervous (pathological)
TOS_He	subscale of the TOS scale, examining the severity of orthorexia, referred to as healthy (nonpathological)

## Appendix A

The Intra- and Interpersonal Effects Scale of Orthorexia (IIESO) by M. Gortat and M. Samardakiewicz.

**Table:**

(PL) Poniższe pytania odnoszą się do Twoich przekonań i zachowań dotyczących żywienia. Chcemy poznać jak ważne jest dla Ciebie przestrzeganie diety. Zaznacz w jakim stopniu zgadzasz się z poniższymi twierdzeniami. Jeśli zaznaczysz 1 będzie to oznaczać, że zupełnie nie zgadzasz się z danym stwierdzeniem, jeśli zaznaczysz 5 oznacza to, że twierdzenie bardzo zgadzasz się z danym stwierdzeniem. (EN) The following questions relate to your beliefs and behaviors regarding nutrition. We want to know how important dietary compliance is for you. Please mark to what extent you agree with the following statements. If you mark 1, it means that you completely disagree with the statement; if you mark 5, it means that you strongly agree with the statement.						
1	(PL) Często podczas rozmów ze znajomymi opowiadam jak ważna jest zdrowa dieta. (EN) Often, during conversations with friends, I discuss how important a healthy diet is.	1	2	3	4	5
2	(PL) Gdy zjem niezdrowy posiłek odmawiam sobie pozostałych posiłków tego dnia nawet gdy są one zdrowe a ja jestem głodna/y. (EN) When I eat an unhealthy meal, I deny myself the rest of the meals that day, even when they are healthy and I am hungry.	1	2	3	4	5
3	(PL) Staram się uświadamiać znajomych i rodzinę o tym, że trzeba dokładnie wybierać produkty do spożycia, aby były zdrowe. (EN) I try to make friends and family realize that it is necessary to carefully choose products to consume in order to be healthy.	1	2	3	4	5
4	(PL) Lubię spożywać posiłki w restauracjach/lokalach. (EN) I like to dine in restaurants/lounges.	1	2	3	4	5
5	(PL) Często zamawiam gotowe posiłki zamiast gotować samemu. (EN) I often order ready-made meals instead of cooking myself.	1	2	3	4	5
6	(PL) Moja wiedza na temat zdrowego odżywiania i szkodliwych substancji w żywności jest duża. (EN) My knowledge about healthy eating and harmful substances in food is high.	1	2	3	4	5
7	(PL) Znajomi uznają mnie za osobę, która ma dużą wiedzę na temat zdrowej żywności. (EN) My friends consider me a person who has a lot of knowledge about healthy food.	1	2	3	4	5
8	(PL) Często nie mogę zasnąć, bo myślę czy posiłki, które spożywam są bezpieczne i zdrowe. (EN) I often cannot sleep because I think about whether the meals I eat are safe and healthy.	1	2	3	4	5
9	(PL) Gdy zjem coś niezdrowego to stosuje diety oczyszczające. (EN) When I eat something unhealthy, I follow a cleansing diet.	1	2	3	4	5
10	(PL) Gdy zdarzy mi się zjeść coś niezdrowego myślę, żeby spowodować wymioty. (EN) When I accidentally eat something unhealthy, I think about causing vomiting.	1	2	3	4	5
11	(PL) Prześladuje mnie poczucie winy, gdy zjem coś niezdrowego. (EN) I am persecuted by guilt when I eat something unhealthy.	1	2	3	4	5
12	(PL) Inni uważają, że przykładam zbyt dużo wagi zdrowemu stylowi życia i odżywianiu się (EN) Others think I place too much importance on a healthy lifestyle and nutrition.	1	2	3	4	5
13	(PL) Często spożywam posiłki przygotowane przez inne osoby. (EN) I often eat meals prepared by others.	1	2	3	4	5
14	(PL) Mam wrażenie, że znajomi unikają ze mną rozmów na temat zdrowego odżywiania się. (EN) I have the impression that my friends avoid talking to me about healthy eating.	1	2	3	4	5
15	(PL) Uważam, że inni nie przykładają odpowiedniej uwagi do jakości i bezpieczeństwa produktów które spożywamy co może być niebezpieczne dla zdrowia. (EN) I believe that others do not pay adequate attention to the quality and safety of the products that we consume, which can be dangerous to out health.	1	2	3	4	5
16	(PL) Jestem zdrowy, bo unikam żywności zawierającej konserwanty i sztuczne barwniki. (EN) I am healthy because I avoid foods containing preservatives and artificial colors.	1	2	3	4	5
17	(PL) Żywność jest złej jakości, dlatego trzeba zażywać suplementy diety. (EN) Food is of poor quality, so dietary supplements must be taken.	1	2	3	4	5
18	(PL) Podczas spotkań ze znajomymi wymieniamy się wiedzą na temat szkodliwości niektórych pokarmów. (EN) During meetings with friends, we exchange knowledge about the harmfulness of certain foods.	1	2	3	4	5
19	(PL) Poświęcam dużo uwagi planowaniu i przygotowywaniu posiłków. (EN) I pay a lot of attention to meal planning and preparation.	1	2	3	4	5
20	(PL) Nie przykładam większej uwagi do spożywanych posiłków. (EN) I do not pay much attention to the meals I eat.	1	2	3	4	5

(PL)—content in Polish; (EN)–content in English.

(PL) Instrukcja obliczania wyników

Należy odwrócić wyniki w itemach: 4; 5; 13; 20.
Dla uzyskania wyniku w poszczególnych skalach należy zsumować odpowiadajcie im itemy:
INTER = 2 + 6 + 8 + 9 + 10 + 11 + 16 + 17 + 19 + 20R
INTRA = 1 + 3 + 4R + 5R + 7 + 12 + 13R + 14 + 15 + 18
R—wynik odwrócony
Wynik Ogólny stanowi sumę wyników uzyskanych w skali INTER i INTRA.

(EN) Scoring instructions

Reverse the item 4, 5, 13, and 20.
To get a score on each scale, the corresponding items must be added together:
INTER = 2 + 6 + 8 + 9 + 10 + 11 + 16 + 17 + 19 + 20R
INTRA = 1 + 3 + 4R + 5R + 7 + 12 + 13R + 14 + 15 + 18
R—reverse item
The overall score is the sum of the scores obtained on the INTER and INTRA scales.
